# Unveiling Genetic Variation in the Seed Bug *Spilostethus pandurus* (Scopoli, 1763) (Hemiptera: Lygaeidae) in Thailand Using Mitochondrial *CO1* Sequence

**DOI:** 10.3390/biology14081022

**Published:** 2025-08-08

**Authors:** Warayutt Pilap, Nakorn Pradit, Chavanut Jaroenchaiwattanachote, Jatupon Saijuntha, Watee Kongbuntad, Wittaya Tawong, Chairat Tantrawatpan, Weerachai Saijuntha

**Affiliations:** 1 Walai Rukhavej Botanical Research Institute, Mahasarakham University, Maha Sarakham 44150, Thailand; warayutt@msu.ac.th (W.P.); nakorn.p@msu.ac.th (N.P.); 2 Center of Excellence in Biodiversity Research, Mahasarakham University, Maha Sarakham 44150, Thailand; chavanut.j@msu.ac.th; 3 Faculty of Engineering, Mahasarakham University, Maha Sarakham 44150, Thailand; jatupons2534@gmail.com; 4 Program in Biotechnology, Faculty of Science, Maejo University, Chiang Mai 50290, Thailand; watee@mju.ac.th; 5 Department of Agricultural Sciences, Faculty of Agriculture Natural Resources and Environment, Naresuan University, Phitsanulok 65000, Thailand; wittayat@nu.ac.th; 6 Center of Excellence in Biodiversity, and Center of Excellence in Research for Agricultural Biotechnology, Naresuan University, Phitsanulok 65000, Thailand; 7 Division of Cell Biology, Department of Preclinical Sciences, Faculty of Medicine, and Center of Excellence in Stem Cell Research and Innovation, Thammasat University, Rangsit Campus, Pathum Thani 12120, Thailand; 8 Biomedical Science Research Unit, Faculty of Medicine, Mahasarakham University, Maha Sarakham 44000, Thailand

**Keywords:** crown flower, pest insects, biodiversity, genetic diversity, haplotype network, phylogenetic tree

## Abstract

*Spilostethus pandurus* is a phytophagous insect widely distributed across Asia, Europe, and Africa, yet its genetic variation remains poorly understood. This study presents the first comprehensive analysis of the genetic diversity and structure of *S. pandurus* in Thailand using mitochondrial cytochrome c oxidase subunit 1 (*CO1*) sequences from 202 individuals across 27 localities. A total of 58 haplotypes were identified, with high haplotype and nucleotide diversity observed, suggesting substantial genetic variation. The haplotype network revealed a star-like topology, indicating recent population expansion or ongoing gene flow. Neutrality tests and mismatch distribution analyses showed no strong signal of recent demographic expansion. Phylogenetic analysis confirmed that all Thai specimens clustered within a well-supported *S. pandurus* clade along with sequences from India, Namibia, and Europe. Analysis of Molecular Variance (AMOVA) revealed significant genetic differentiation among four continental groups, indicating that geographic isolation and restricted gene flow have shaped genetic divergence at a broad biogeographic scale. Further research using highly polymorphic nuclear markers is recommended to better resolve the population structure and evolutionary history of *S. pandurus* in Thailand and beyond.

## Simple Summary

This study provides the first comprehensive insight into the genetic variation of the seed bug *Spilostethus pandurus* in Thailand, an insect of agricultural importance. Using mitochondrial *CO1* gene sequences from 202 individuals across 27 locations, we uncovered high genetic diversity with 58 haplotypes, many of which were unique to specific areas. These findings highlight the species’ adaptability and potential for local differentiation. The low divergence from populations in other continents suggests ongoing gene flow. This research offers a valuable foundation for future studies on pest management, population dynamics, and conservation of phytophagous insects.

## 1. Introduction

Understanding the genetic variation and genetic structure of insect pest species is essential for unraveling their evolutionary dynamics and developing effective management strategies [1]. In Thailand, a variety of insects act as pests of economically and ecologically valuable plants, including weevils, aphids, fruit flies, bugs, and beetles [2]. Among these, the seed bug *Spilostethus pandurus* (Scopoli, 1763) (Hemiptera: Lygaeidae) is a prominent phytophagous pest that damages crown flower plants and other host species [3]. *S. pandurus* is widely distributed across tropical and subtropical regions and is well known for its aposematic coloration and ability to sequester toxic compounds from plants such as milkweeds, which provide chemical defense against predators [4]. This species exemplifies both Müllerian mimicry and automimicry, making it a valuable model for exploring ecological adaptation, chemical defense, and insect behavior [5].

Currently, five subspecies of *Spilostethus pandurus* (Scopoli, 1763) are recognized based on morphological characteristics and geographic distribution [6]: *S. p. pandurus*, *S. p. militaris* (Fabricius, 1775), *S. p. asiaticus* (Kolenati, 1845), *S. p. elegans* (Wolff, 1802), and *S. p. tetricus* (Horváth, 1909) [6]. These subspecies differ primarily in color patterns and regional occurrence. *S. p. pandurus* is distributed in Europe, North Africa, and the Middle East and exhibits the typical bright red and black coloration. In contrast, *S. p. militaris*, found across South and Southeast Asia (e.g., India, Thailand, Indonesia), is characterized by yellowish or orange-red coloration. *S. p. asiaticus*, with a less well-defined range in parts of Asia, shows intermediate coloration between red and orange with black markings. *S. p. elegans*, native to tropical Africa, is noted for its vivid and contrasting color patterns, while *S. p. tetricus*, also endemic to Africa, is distinguished by a violaceous (purplish) sheen on parts of the body [6]. Despite these morphological distinctions, the taxonomic status and phylogenetic relationships among these subspecies remain underexplored, particularly in Asia, where subspecific boundaries may be unclear.

Although previous research has provided insights into the behavioral ecology of *S. pandurus*, including its feeding habits, social behavior, and reproductive strategies, the genetic underpinnings of these traits remain poorly understood. Notably, earlier work using RAPD markers revealed significant genetic differentiation among populations of *S. pandurus* in South Sinai, Egypt, even over short geographic distances, suggesting that habitat fragmentation can limit gene flow [7]. Yet, despite its wide distribution and ecological importance, comprehensive genetic data for this species across its broader range remain scarce. Investigating its genetic variation can offer insights into its evolutionary history, gene flow, and population structure, which are key factors for understanding its adaptability to environmental change and for guiding future management and control efforts [8].

Mitochondrial DNA (mtDNA) has proven to be a valuable tool in genetic research, particularly for detecting genetic variation within and among species [9]. Among mitochondrial genes, cytochrome c oxidase subunit 1 (*CO1*) is widely used as a genetic marker in population genetics and phylogeographic studies due to its relatively high mutation rate, maternal inheritance, and lack of recombination [10]. The *CO1* gene is central to DNA barcoding, where it assists in species identification, evaluation of genetic diversity, and reconstruction of evolutionary relationships [11]. In the case of *S. pandurus*, *CO1* sequence analysis can uncover genetic differences among geographically isolated populations, providing insights into gene flow, migration dynamics, and historical biogeography. Previous studies successfully examined genetic variation and revealed the cryptic species of several pests and edible insects in Thailand using *CO1* sequences [12,13,14].

Through this research, we aim to investigate the genetic variation of *S. pandurus* in Thailand using the mitochondrial *CO1* gene as a molecular marker. This approach aims to evaluate intraspecific genetic diversity and assess genetic differentiation among populations from distinct geographic regions. The findings will contribute to a deeper understanding of the population genetics and evolutionary history of *S. pandurus*. Furthermore, this study adds to the growing body of literature utilizing mitochondrial markers to explore genetic variation in insect populations, thereby enhancing our understanding of genetic diversity across species and ecosystems.

## 2. Materials and Methods

### 2.1. Sample Collection and Molecular Analysis

**Figure 1:**
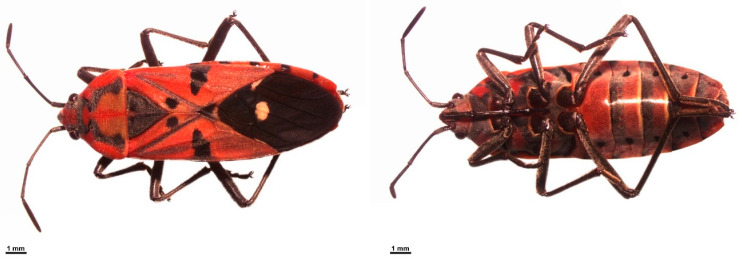
Adult seed bug *Spilostethus pandurus* shown in dorsal and ventral views.

**Figure 2:**
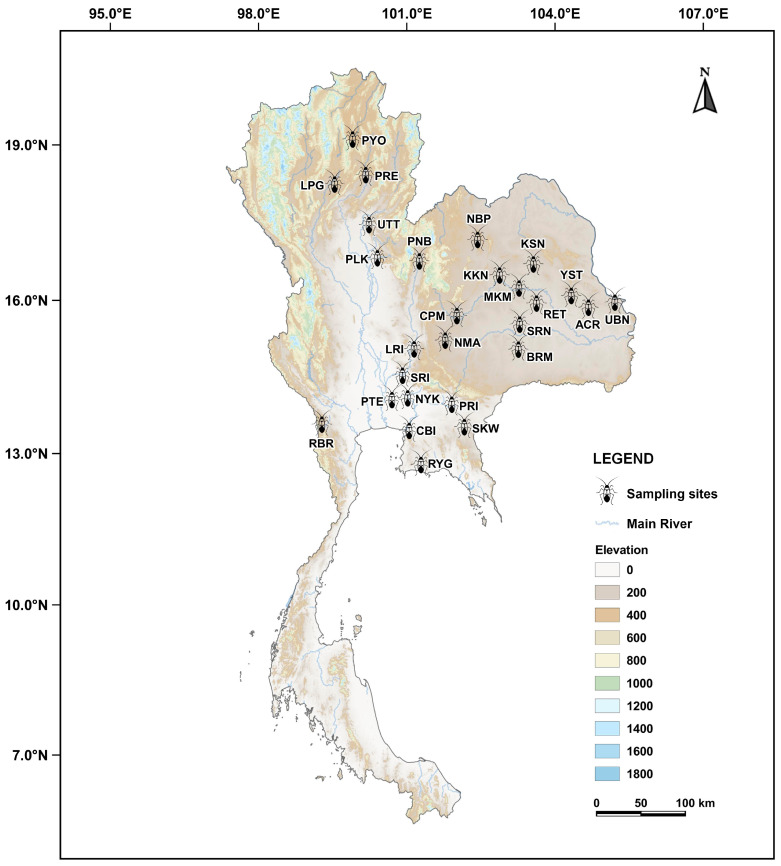
Map indicating the sampling localities of *Spilostethus pandurus* populations across Thailand. Each marked of locality code corresponds to a listed in Table 1.

A total of 202 seed bugs, *S. pandurus* (Figure 1), were collected by hand-picking from their host plant, *Calotropis* spp., at 27 localities across Thailand (Table 1 and Figure 2). The specimens were immobilized by chilling on ice for approximately 10 min, then immediately preserved in 80% ethanol. Species identification was carried out based on morphology using the identification key for Iberian Lygaeinae provided by Vivas [15]. Total DNA was individually extracted from the left foreleg of each seed beetle using E.Z.N.A.^®^ Tissue DNA kit (Omega bio-tek, Norcross, GA, USA) following the manufacturer’s protocol. DNA samples were kept at −20 °C for further molecular analysis. A portion of the *CO1* fragment was amplified and sequenced following the primers and PCR conditions described by Pradit et al. [12]. The PCR products were electrophoresed in 1% agarose gels and visualized with GelRed^TM^ Nucleic Acid Gel Stain (Biotium, Inc., Hayward, CA, USA). The amplified band was cut and purified by using E.Z.N.A.^®^ Gel Extraction kit (Omega bio-tek, USA). The purified PCR products were sent for DNA sequencing at ATGC Co., Ltd., Khlong Luang, Pathum Thani, Thailand.

### 2.2. DNA Sequence Analyses

All *CO1* sequences generated in this study were aligned using the ClustalW program version 2.0 [16] and compared the variable sites between haplotypes in the BioEdit program version 7.2.5 [17]. Molecular diversity indices, haplotype data, and mismatch distribution analysis were calculated using the DnaSp v5 program [18]. The genetic difference between populations within a species and between different species of the genus *Spilostethus* was calculated based on *p*-distance and Kimura 2-parameter (K2P) distance [19] using the program MEGA XI [20]. A minimum-spanning haplotype network was constructed in the Network program version 10.2 (https://www.fluxus-engineering.com/; accessed on 28 May 2025) based on a median-joining network [21] using all sequences generated in this study. Neutrality tests, including Tajima’s D and Fu’s Fs, genetic differentiation (Φ_ST_) analyses, were conducted using the Arlequin program version 3.5.2.2 [22]. Genetic differentiation among populations was assessed using Analysis of Molecular Variance (AMOVA) implemented in Arlequin version 3.5.2.2 [22]. The analysis was used to partition genetic variation at different hierarchical levels, and to estimate the following fixation indices: *F*_CT,_ which quantifies genetic variation among predefined groups; *F*_SC,_ which estimates variation among populations within groups; and *F*_ST,_ which measures overall genetic differentiation among all populations. Significance of the variance components and fixation indices was tested using 10,000 permutations, and results with *p*-values < 0.05 were considered statistically significant.

### 2.3. Phylogenetic Tree Reconstruction

Phylogenetic trees were constructed using *CO1* sequences of *S. pandurus* generated in this study along with *CO1* sequences of other *Spilostethus* species retrieved from GenBank. The inclusion of multiple *Spilostethus* species aimed to provide a comparative framework for evaluating the genetic distinctiveness and phylogenetic placement of *S. pandurus* within the genus. The *CO1* sequences of *Kleidocerys resedae* (Panzer, 1797), *Parapiesma quadratum* (Fieber, 1844), and *Henesaris laticeps* (Curtis, 1837) were used as the outgroup. Maximum likelihood (ML) phylogenetic analysis was performed using the General Time Reversible model with gamma distribution and invariant sites (GTR + G + I model) [23], while neighbor-joining (NJ) trees [24] were also constructed. Both analyses were conducted using MEGA XI [20], with nodal support estimated by 1000 bootstrapping replicates. Using the corrected Akaike information criterion (AIC), MrModeltest 2.4 [25] provided the best-fit evolutionary models for the *CO1* sequence. The GTR + I + G was shown to be the best-fit model for phylogenetic analysis. For the Bayesian Inference (BI) study, the following parameters were set in Mesquite v.3.81 [26]: nst = 6, rates = invgamma. MrBayes v.3.1.2 [27] with Markov chain Monte Carlo (MCMC) was used for BI. With sampling every 10,000 generations, two parallel analyses were conducted for 30 million generations, each involving four chains. The analyses continued until the split frequencies’ average standard deviation remained below 0.01. The ESS values datasets were used to evaluate run convergence using Tracer 1.7.2 [28]. With the first 25% of trees discarded as burn-in, Bayesian posterior probabilities were calculated for a 50% majority rule consensus tree of the remaining trees.

### 2.4. Species Delimitation Analysis

Two single-locus species delimitation methods, namely Assemble Species by Automatic Partitioning; ASAP [29] and Poisson Tree Processes; mPTP [30] were applied to *CO1* marker for genetic lineage delineation of both species. ASAP analysis was performed using the online tool (https://bioinfo.mnhn.fr/abi/public/asap/; accessed on 28 May 2025). The species partition with the lowest ASAP score and a suitable threshold distance (dT) was selected under the Kimura (K80) model. Default parameters were used, with a transition/transversion ratio (ts/tv) of 2.0 and a minimum and maximum threshold distance of 0.05 to 0.5. The mPTP analysis was conducted using a web server (https://mptp.h-its.org/#/tree/; accessed on 28 May 2025). The ML trees for the *CO1* genes generated in MEGA XI [20], were used as input files. All parameters were kept at default, employing a single threshold (*p* = 0.001) for delimitation.

## 3. Results

### 3.1. Genetic Diversity Analyses

Mitochondrial *CO1* sequences of *S. pandurus* generated in this study were deposited in GenBank under accession numbers PV648460–PV648517. A total of 202 individuals from 27 localities were analyzed. Overall, genetic diversity was high, with 54 variable sites detected, comprising 14 singleton variable sites and 40 parsimony-informative sites (Table S1). Based on these variations, 58 haplotypes (Sp1–Sp58) were identified. Haplotype diversity (Hd) ranged from 0.000 ± 0.000 to 1.000 ± 0.272, with an average of 0.906 ± 0.013, while nucleotide diversity (Nd) ranged from 0.0000 ± 0.0000 to 0.0078 ± 0.0025, with an average of 0.0053 ± 0.0003 (Table 2). The highest haplotype diversity was observed in KKN and KSN, with values of 1.000 ± 0.096 and 1.000 ± 0.272, respectively, suggesting rich haplotypic composition. In contrast, due to limited sample sizes, some populations, such as ACR, NMA, and SKW, exhibited no genetic diversity.

### 3.2. Neutrality Tests and Demographic Analyses

**Figure 3:**
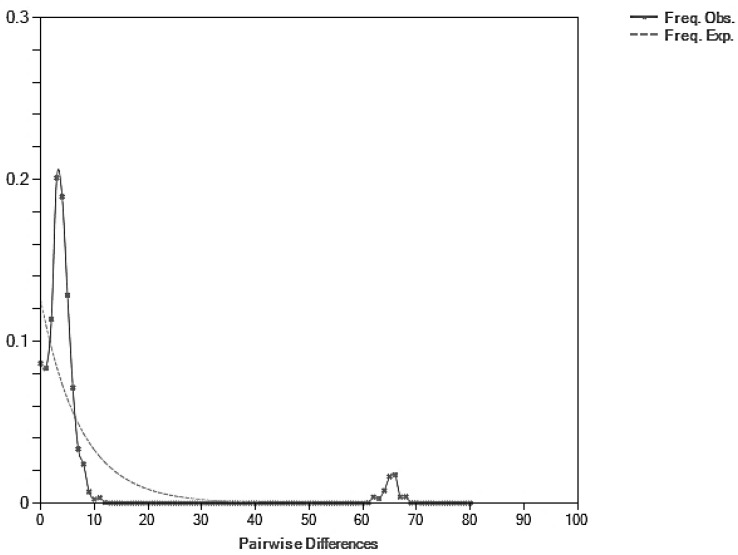
Mismatch distribution graph of *Spilostethus pandurus* based on *CO1* sequence data. The solid line represents the observed frequency of pairwise nucleotide differences, while the dashed line indicates the expected distribution under a model of sudden demographic expansion.

Neutrality tests using Tajima’s D and Fu’s Fs (Table 2) revealed signatures of demographic events. Several populations showed negative and significant values indicating potential population expansion or purifying selection, such as MKM, UBN, KKN, BRM, and PLK (Table 2). These deviations from neutrality suggest non-random evolutionary processes at work. In contrast, some populations such as NYK and CBI showed positive Fu’s Fs values, which could suggest population contraction or balancing selection, although these were not statistically significant. The mismatch distribution analysis based on mitochondrial *CO1* sequences revealed a multimodal distribution (Figure 3), which is typically indicative of a population at demographic equilibrium or one that has experienced long-term population stability rather than a recent expansion. The observed distribution did not fit the expected unimodal curve under a sudden expansion model. This inference is supported by the results of neutrality tests. Tajima’s D value was −0.6045 and Fu’s Fs was −0.3471, both of which were non-significant (Table 2), indicating no strong evidence of demographic expansion.

### 3.3. Genetic Differences, Genetic Structure, and Haplotype Analyses

**Figure 4:**
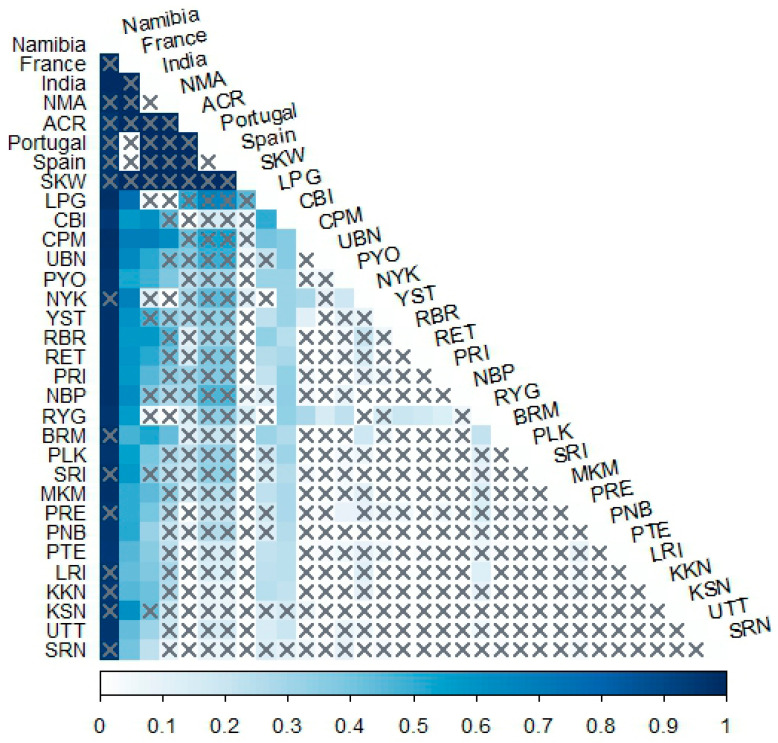
Heat map showing genetic difference represented by Φ_ST_ values based on *CO1* sequences among populations of *Spilostethus pandurus* from Thailand and other countries. The x-axis represents Φ_ST_ values ranging from 0 to 1, corresponding to variation in color shedding. The y-axis represents the locality codes of *S. pandurus* populations. Cross marks (x) indicate no significant difference (*p*-value ≥ 0.05), while other values represent significant genetic differences (*p*-value < 0.05). Locality codes are provided in Table 1.

Pairwise genetic distances among *S. pandurus* populations from Thailand and other countries retrieved from GenBank were estimated using both *p*-distance (lower triangle) and Kimura 2-parameter (K2P) model (upper triangle) (Table S2). The *p*-distances ranged from 0.0000 to 0.0103, while K2P distances ranged from 0.0000 to 0.0104. The smallest genetic distance (0.0000) was observed between populations NMA and India. The highest distances for both models were found between the Namibian population and populations KKN, NMA, RYG, and NYK, suggesting that the Namibian population from the African continent is the most genetically distinct (Table S2). Genetic difference (Φ_ST_) analysis revealed many populations exhibit low or non-significant differentiation, indicating possible gene flow or shared ancestry. However, some populations, particularly CBI, RYG, and LPG, were genetically distinct from the others (Figure 4). Overall, the data show varying degrees of genetic differentiation among *S. pandurus* populations in Thailand. Notably, the Namibian, French, and Indian populations showed significant differentiation from most Thai populations, reinforcing their genetic distinctiveness.

**Figure 5:**
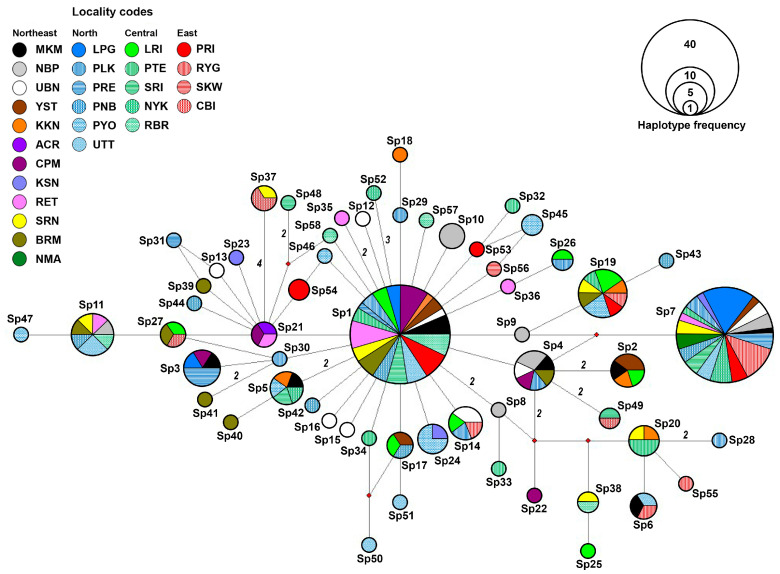
Minimum spanning haplotype network constructed from *CO1* haplotypes of *Spilostethus pandurus* populations in Thailand. Each color and pattern represent a different sampling locality (see Table 1 for details). The size of each circle corresponds to the number of individuals sharing that haplotype. Numbers on the branches indicate the number of mutational steps between haplotypes; branches without numbers represent a single mutational step.

The haplotype network constructed from mitochondrial *CO1* sequences revealed a total of 58 haplotypes (Sp1–Sp58) among 202 individuals from 27 localities across Thailand (Figure 5). Unfortunately, we were unable to include the sequences from GenBank in the haplotype analysis because most of them are shorter than the sequences generated in this study, making them unsuitable for accurate haplotype reconstruction. The network displayed a star-like topology centered around a few predominant haplotypes, notably Sp1 and Sp7. These common haplotypes were widely shared among multiple populations across all four geographic regions (northeast, north, central, and east), indicating potential ancestral lineages and suggesting a pattern of recent population expansion or ongoing gene flow. A large number of haplotypes (39 of 58; 67.2%) were unique to a single locality, reflecting a high level of localized genetic differentiation of *S. pandurus* populations in Thailand. The number of mutational steps separating haplotypes ranged from one to four, with several haplotypes positioned on long branches, suggesting sequence divergence from central haplotypes. One haplotype (Sp27), shared between samples from SRN in the northeast and CBI in the eastern regions, was the most distinct, differing from the others by four mutational steps. Despite this, most haplotypes were closely connected, differing by only one or two mutational steps, indicating low overall genetic divergence.

Analysis of Molecular Variance (AMOVA) of the genetic structure of *S. pandurus* populations, based on *CO1* sequences, demonstrated significant genetic differentiation among the four defined groups, namely, Thai, European, South African (Namibia), and Asian groups, with *F*_CT_ = 0.85414 (*p*-value < 0.001). Additionally, significant genetic variation was detected among populations within groups (*F*_SC_ = 0.04483, *p*-value < 0.001) and among individuals within populations (*F*_ST_ = 0.86068, *p*-value < 0.001) (Table 3). These results indicate that genetic differentiation is most pronounced between groups, with moderate differentiation among populations within groups and substantial variation within populations.

### 3.4. Phylogenetic Tree and Species Delimitation Analyses

**Figure 6:**
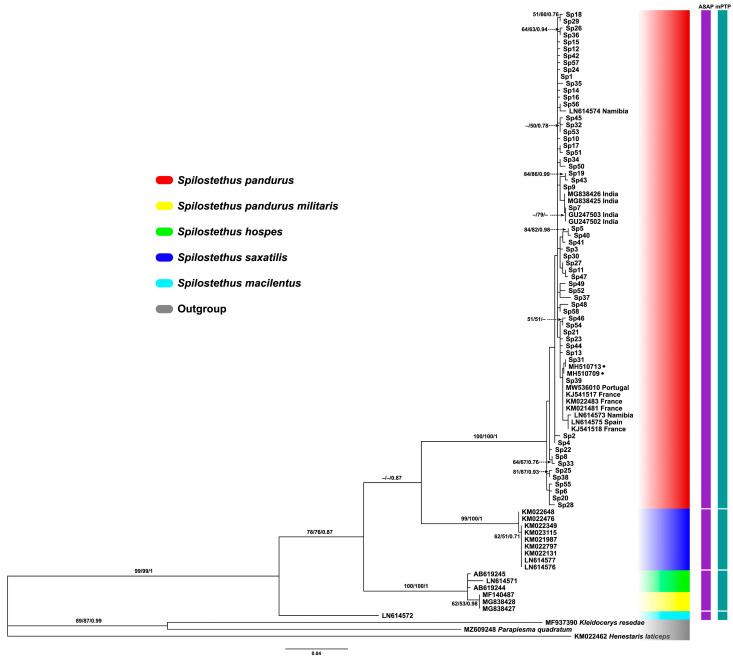
Phylogenetic tree constructed from *CO1* sequences of seed bug genus *Spilostethus*. Each color bar represents a different clade of different species. Bootstrap values for maximum likelihood and neighbor-joining, as well as posterior probabilities for Bayesian Inference (BI), are indicated above or near the branches. The scale bar represents 0.04 substitutions per nucleotide position. Each colored bar represents a species delimitation method: the purple bar indicates the Assemble Species by Automatic Partitioning (ASAP), and the green bar denotes Poisson Tree Processes (mPTP) methods. Different genetic groups are indicated by gaps within each bar. The symbol • indicates that the taxon represents a sample of unknown origin.

The phylogenetic tree reconstructed from mitochondrial *CO1* sequences clearly delineated four distinct clades of the genus *Spilostethus* with *K. resedae*, *P. quadratum*, and *H. laticeps* used as the outgroup (Figure 6). All haplotypes identified in this study (Sp1–Sp58) clustered within the well-supported clade (red shaded), along with published sequences from India, Namibia, Portugal, France, and Spain. This clade showed moderate to high support and included both shared and locality-specific haplotypes, confirming the identity of the studied specimens as *S. pandurus*, but they could not be confidently classified into any subspecies. Several Thai haplotypes (e.g., Sp1, Sp7, Sp21, Sp3) grouped closely with reference sequences from geographically distant populations, such as India in Asia, Namibia in Africa, Portugal, France, and Spain in Europe, indicating low intercontinental divergence. Two other species, *S. saxatilis* (Scopoli, 1763) and *S. macilentus* (Stål, 1874) formed well-separated clades, supported by ML, NJ, and BI analyses, as well as by species delimitation methods (ASAP and mPTP), whereas *S. pandurus militaris* (Fabricius, 1775) and *S. hospes* (Fabricius, 1794) were grouped as a single clade (Figure 6).

## 4. Discussion

As far as we are aware, research on the genetic variation of the seed bug *S. pandurus* at the global level is scarce. To date, only a single study has documented notable genetic differentiation among *S. pandurus* populations in Egypt based on RAPD analysis [7]. Consequently, this study is the first to provide a detailed assessment of the genetic variation of *S. pandurus* in Thailand. Our results revealed considerable genetic diversity, characterized by high genetic and haplotype diversity indexes. These findings indicate that natural populations of *S. pandurus* in the surveyed regions possess substantial genetic diversity, likely reflecting active gene flow and a pattern consistent with recent population expansion or continued genetic exchange. Comparable results have been observed in various pest and edible insect species in Thailand, such as the giant water bug *Lethocerus indicus* (Lepeletier and Serville, 1825) [12], mole cricket *Gryllotalpa orientalis* (Latreille, 1802) [13], and jewel beetles *Sternocera aequisignata* (Saunders, 1866) and *S. ruficornis* (Saunders, 1866) [14].

However, our results revealed no significant genetic sub-structuring associated with geographic distance among *S. pandurus* populations within Thailand. In contrast, a previous study using RAPD markers reported significant genetic differentiation among *S. pandurus* populations in South Sinai and Egypt [7]. This discrepancy may stem from differences in the molecular markers employed. RAPD markers are capable of detecting more recent or fine-scale genetic variation, while the mitochondrial *CO1* gene used in our study is maternally inherited, evolves relatively slowly, and may lack the resolution needed to detect subtle population structure at a local scale. Therefore, the *CO1* marker may be limited in its ability to uncover fine-scale genetic differentiation and population structure in *S. pandurus*. To gain deeper insights into the genetic diversity, population dynamics, and evolutionary history of this species, future studies should consider incorporating highly polymorphic nuclear markers, such as non-coding intron regions, microsatellites, or single nucleotide polymorphisms (SNPs). Nevertheless, our findings did reveal significant genetic sub-structuring among populations from different continents, including Thailand, Europe, southern Africa (Namibia), and other parts of Asia, suggesting that geographic isolation and limited gene flow have shaped the genetic divergence of *S. pandurus* at a broader biogeographic scale.

The low genetic structure observed among *S. pandurus* populations in Thailand may be attributed to high levels of gene flow, possibly facilitated by the dispersal ability of *Spilostethus* bugs and the absence of strong geographic barriers. However, it is also important to consider the potential influence of maternally inherited endosymbionts such as *Wolbachia*. This bacterial symbiont is known to manipulate host reproduction and can lead to cytoplasmic hitchhiking, which may obscure signals of population differentiation in mitochondrial DNA. Notably, *Wolbachia* infection has already been documented in several species within the genus *Spilostethus*, including *S. pandurus* [31], suggesting that its presence might contribute to the observed genetic patterns. Although the current study did not screen for *Wolbachia* infection, future research should include diagnostic assays for endosymbionts to clarify their potential role in shaping host genetic structure.

Phylogenetic tree and species delimitation test reveal that *S. hospes*, *S. saxatilis*, and *S. macilentus*, were clearly clustered as distinct species clades, except for the subspecies *S. pandurus militaris*, which clustered within the same clade as *S. hospes*, a pattern also supported by the species delimitation results. As previously mentioned, *S. pandurus* includes five subspecies distinguished based on morphological characteristics and geographic distribution [6]. However, there is limited understanding of the morphological variation, taxonomic validity, and phylogenetic relationships among these subspecies. Therefore, our findings possibly suggest that either *S. pandurus militaris* or *S. hospes* may have been misidentified or may not represent valid, distinct taxa. This also raises important questions about the composition of the *S. pandurus* clade (indicated as “red clade” in Figure 6), specifically whether it corresponds to the nominative subspecies *S. pandurus pandurus* or represents a group of multiple subspecies. Clarifying this distinction is essential for interpreting the observed phylogenetic patterns and understanding the evolutionary relationships within this complex species group. Further analyses should be conducted by incorporating a broader geographic sampling of each subspecies of *S. pandurus* individuals, with accurate subspecies identification, along with detailed morphological comparisons and integrative taxonomic approaches combining molecular, morphological, and ecological data.

Interestingly, we observed a high proportion of unique haplotypes within each population of *S. pandurus* in Thailand, which may reflect local adaptation to specific environmental conditions. Such genetic differentiation is often driven by selective pressures unique to each habitat, such as variations in climate, host plant availability, or predation pressures, leading to population-specific evolutionary trajectories. Similar patterns of localized genetic divergence due to environmental adaptation have been reported in other phytophagous insects [32]. In addition to environmental adaptation, historical demographic events, such as population bottlenecks, founder effects, and subsequent population expansions, could have contributed to the observed genetic pattern. These events can reduce genetic variation temporarily, followed by the rapid accumulation of novel mutations within isolated populations [33].

One important ecological factor to consider is the host plant association. *S. pandurus* is known to be as a pest of various plant species, showing a strong preference for crown flower trees [3]. If *S. pandurus* exhibits similar host specificity or dependence, differences in local host plant availability and quality could drive selective pressures unique to each region, promoting the emergence and maintenance of population-specific haplotypes. Such host-associated differentiation (HAD) has been well documented in phytophagous insects, where adaptation to different host plants can lead to genetic divergence even in the absence of geographic isolation [34]. Additionally, if populations are locally specialized on distinct plant chemotypes or microhabitats, this could reinforce reproductive isolation and restrict gene flow.

Moreover, the pest status of *S. pandurus* indicates frequent interaction with human-modified environments, such as agricultural and urban areas, which can impose additional selective pressures (e.g., pesticide exposure, habitat disturbance). These anthropogenic factors may accelerate microevolutionary processes and promote haplotype differentiation [35]. Combined with limited dispersal ability, habitat fragmentation, and historical demographic events, these dynamics likely contribute to the high proportion of unique haplotypes observed in *S. pandurus* populations. This is consistent with a previous study that found notable genetic differentiation among *S. pandurus* populations in South Sinai, Egypt, despite their close geographic proximity [7].

The genetic insights gained from this study have important implications for the management of *S. pandurus* as a pest species. The high haplotype diversity and presence of population-specific haplotypes suggest that local populations may respond differently to control strategies, such as insecticide applications or host plant resistance [32,34]. The lack of clear geographic structuring further implies that pest populations may disperse widely or recolonize treated areas, potentially undermining localized control efforts [33]. Moreover, the influence of environmental and anthropogenic factors on genetic differentiation highlights the need for integrated pest management (IPM) strategies that consider both ecological and evolutionary dynamics [1,8]. Monitoring genetic variation over time can help detect shifts in population structure resulting from selection pressures imposed by control methods.

## 5. Conclusions

This study provides the first comprehensive assessment of the genetic variation of *Spilostethus pandurus* in Thailand, revealing high haplotype diversity across populations but no significant genetic sub-structuring associated with geographic localities. However, comparisons among four broader population groups (Thailand, Europe, Namibia, and other parts of Asia) suggest that geographic isolation and limited gene flow have contributed to the genetic divergence of *S. pandurus* at a continental scale. In the future, the application of more variable nuclear markers, such as nuclear introns, microsatellites, and SNPs, may offer deeper insights into the genetic diversity, population structure, and evolutionary history of *S. pandurus* within Thailand and across its global distribution.

## Figures and Tables

**Table 1:** Sampling localities and other related details for *Spilostethus pandurus* populations collected in Thailand.

Code	*n*	District	Province	Coordinates	Region
MKM	9	Mueang	Maha Sarakham	16°14′42.6″ N, 103°16′23.6″ E	Northeast
NBP	10	Mueang	Nong Bue Lumphu	17°11′07.6″ N, 102°26′05.7″ E	Northeast
UBN	10	Khemarat	Ubon Ratchathani	15°58′12.9″ N, 105°12′47.3″ E	Northeast
YST	6	Kut Chum	Yasothon	16°01′04.5″ N, 104°20′11.2″ E	Northeast
KKN	6	Mueang	Khon Kaen	16°29′51.6″ N, 102°52′48.8 ″E	Northeast
ACR	1	Mueang	Amnat Charoen	15°52′14.0″ N, 104°40′40.1″ E	Northeast
CPM	8	Mueang	Chaiyaphum	15°42′30.0″ N, 102°00′53.0″ E	Northeast
KSN	3	Sahatsakhan	Kalasin	16°42′41.2″ N, 103°33′50.6″ E	Northeast
RET	9	Mueang	Roi Et	15°58′56.9″ N, 103°35′57.8″ E	Northeast
SRN	9	Chompon Buri	Surin	15°22′14.3″ N, 103°16′19.6″ E	Northeast
BRM	10	Satuek	Buri Ram	15°15′49.4″ N, 103°15′54.2″ E	Northeast
NMA	2	Dan Khun Thot	Nakhon Ratchasima	15°13′43.7″ N, 101°46′44.7″ E	Northeast
LPG	10	Mueang	Lampang	18°15′14.8″ N, 99°32′25.5″ E	North
PLK	10	Wang Thong	Phitsanulok	16°49′51.9″ N, 100°24′25.0″ E	North
PRE	7	Song	Phrae	18°26′04.4″ N, 100°10′13.2″ E	North
PNB	9	Lom Sak	Phetchabun	16°46′09.4″ N, 101°15′19.8″ E	North
PYO	10	Mueang	Phayao	19°06′56.6″ N, 99°54′17.7″ E	North
UTT	10	Mueang	Uttaradit	17°28′22.1″ N, 100°14′15.0″ E	North
LRI	10	Tha Luang	Lop Buri	15°03′33.5″ N, 101°09′00.1″ E	Central
PTE	9	Khlong Luang	Pathum Thani	14°03′59.0″ N, 100°42′03.1″ E	Central
SRI	8	Mueang	Saraburi	14°32′33.4″ N, 100°54′54.3″ E	Central
NYK	5	Ongkharak	Nakhon Nayok	14°05′56.6″ N, 101°01′12.5″ E	Central
RBR	7	Suan Phueng	Ratchaburi	13°35′00.3″ N, 99°14′22.6″ E	Central
PRI	10	Kabin Buri	Prachin Buri	13°57′53.8″ N, 101°51′27.2″ E	East
RYG	9	Mu-Mueang	Rayong	12°40′33.3″ N, 101°17′12.6″ E	East
SKW	1	Watthana Nakhon	Sa Kaeo	13°31′59.6″ N, 102°09′59.0″ E	East
CBI	4	Phanat Nikhom	Chon Buri	13°27′36.7″ N, 101°02′59.3″ E	East
Total	202				

*n*, sample size.

**Table 2:** Molecular diversity indices and neutrality test results for *Spilostethus pandurus* from different geographical localities in Thailand, based on *CO1* sequence analysis.

Populations	Molecular Diversity Indices						Neutrality Test	
	*n*	S	H	Uh	Hd ± SD	Nd ± SD	Tajima’s D	Fu’s Fs
MKM	9	14	7	0	0.917 ± 0.092	0.0057 ± 0.0014	−1.5903 *	−2.1655
NBP	10	8	6	3	0.889 ± 0.075	0.0041 ± 0.0006	−0.2799	−1.1223
UBN	10	9	8	4	0.956 ± 0.059	0.0039 ± 0.0006	−0.8920	−4.1589 *
YST	6	6	4	0	0.867 ± 0.129	0.0041 ± 0.0008	0.0848	0.0220
KKN	6	14	6	1	1.000 ± 0.096	0.0076 ± 0.0014	−1.2666	−2.2144 *
ACR	1	0	1	0	0.000 ± 0.000	0.0000 ± 0.0000	0.0000	0.0000
CPM	8	7	5	1	0.786 ± 0.151	0.0030 ± 0.0011	−1.3593	−1.2317
KSN	3	6	3	1	1.000 ± 0.272	0.0062 ± 0.0018	0.0000	0.1335
RET	9	8	6	2	0.833 ± 0.127	0.0033 ± 0.0009	−1.2835	−2.0335
SRN	9	15	7	0	0.944 ± 0.070	0.0074 ± 0.0012	−0.6481	−1.3381
BRM	10	11	8	3	0.933 ± 0.077	0.0047 ± 0.0009	−0.9718	−3.5050 *
NMA	2	0	1	0	0.000 ± 0.000	0.0000 ± 0.0000	0.0000	0.0000
LPG	10	5	3	0	0.511 ± 0.164	0.0028 ± 0.0009	0.0739	1.7262
PLK	10	14	8	3	0.956 ± 0.059	0.0053 ± 0.0014	−1.3922	−3.0952 *
PRE	7	8	4	1	0.810 ± 0.130	0.0053 ± 0.0011	0.2627	0.9276
PNB	9	11	7	3	0.944 ± 0.070	0.0052 ± 0.0009	−0.8291	−2.0807
PYO	10	13	6	3	0.889 ± 0.075	0.0057 ± 0.0009	−0.9416	−0.3538
UTT	10	13	6	2	0.889 ± 0.075	0.0060 ± 0.0011	−0.7435	−0.2283
LRI	10	16	8	1	0.956 ± 0.059	0.0062 ± 0.0013	−1.3734	−2.6455
PTE	9	13	7	3	0.944 ± 0.070	0.0066 ± 0.0010	−0.5595	−1.6394
SRI	8	11	5	1	0.857 ± 0.108	0.0053 ± 0.0012	−1.0001	−0.0273
NYK	5	8	3	1	0.700 ± 0.218	0.0078 ± 0.0025	−0.3817	2.4613
RBR	7	8	5	2	0.857 ± 0.137	0.0041 ± 0.0013	−0.9631	−0.9428
PRI	10	8	5	2	0.822 ± 0.097	0.0039 ± 0.0008	−0.8100	−0.7457
RYG	9	11	5	1	0.722 ± 0.159	0.0063 ± 0.0016	0.0096	0.7196
SKW	1	0	1	1	0.000 ± 0.000	0.0000 ± 0.0000	0.0000	0.0000
CBI	4	8	3	0	0.833 ± 0.222	0.0070 ± 0.0019	0.3090	1.3432
Total	202	54	58	39	0.906 ± 0.013	0.0053 ± 0.0003	−0.6045	−0.3471

*n*, sample size; S, segregation site; H, number of haplotypes; Uh, unique haplotype; Hd, haplotype diversity; Nd, nucleotide diversity; SD, standard deviation; * *p*-value < 0.05. Locality codes are provided in Table 1.

**Table 3:** Analysis of Molecular Variance (AMOVA) based on *CO1* sequences of *Spilostethus pandurus* populations defined by four population groups, namely, Thailand, Europe, Namibia, and other parts of Asia.

Source of Variation	d.f.	Ss	Vc	%Va	Fixation Indices
Among groups	3	214.08	9.06121	85.41	*F*_CT_ = 0.85414 **
Among populations within groups	28	55.004	0.06936	0.65	*F*_SC_ = 0.04483 *
Within populations	182	268.986	1.47794	13.93	*F*_ST_ = 0.86068 **

d.f., degree of freedom; Ss, some of squares; Vc, variance components; %Va, percentage of variation; * *p*-value < 0.01; ** *p*-value < 0.001.

## Data Availability

All data are available upon request.

## Supplementary Materials

The following supporting information can be downloaded at https://www.mdpi.com/article/10.3390/biology14081022/s1, Table S1: Variable nucleotide positions of *CO1* sequences. Table S2: Genetic differences *p*-distance and 16S rDNA calculated based on *CO1* sequences.
